# Discovery of amino acid substitutions in penicillin-binding proteins associated with adaptation to D-Ala-D-Lac in vancomycin-resistant *Enterococcus faecalis*

**DOI:** 10.3389/fcimb.2025.1522114

**Published:** 2025-02-11

**Authors:** Nese Caglayan, Banu Sancak, Zeynep Kanlidere, Tanil Kocagoz

**Affiliations:** ^1^ Department of Medical Biotechnology, Institute of Health Sciences, Acibadem Mehmet Ali Aydinlar University, Istanbul​, Türkiye; ^2^ Department of Medical Microbiology, School of Medicine, Hacettepe University, Ankara, Türkiye; ^3^ Department of Basic Pharmaceutical Sciences, Faculty of Pharmacy, Acibadem Mehmet Ali Aydinlar University, Istanbul​, Türkiye; ^4^ Department of Medical Microbiology, School of Medicine, Acibadem Mehmet Ali Aydinlar University, Istanbul​, Türkiye

**Keywords:** vancomycin-resistant, enterococci, penicillin-binding proteins, PBP, bacterial cell wall, peptidoglycan

## Abstract

The bacterial cell wall, essential for structural integrity, is synthesized with penicillin-binding proteins (PBPs). Vancomycin-resistant enterococci (VRE) evades vancomycin by replacing D-Ala-D-Ala in their cell wall precursors with D-Ala-D-Lac, reducing the drug’s effectiveness. However, how PBPs-which typically use D-Ala-D-Ala as a substrate-adapt to recognize D-Ala-D-Lac remains unclear. Here, we performed Sanger sequencing and alignment of PBP genes from both vancomycin-susceptible and -resistant *E. faecalis* strains to identify mutations, following amplification by PCR. We then applied homology modeling to assess structural impacts of these changes on PBPs and conducted docking studies to investigate ligand-binding interactions. For the first time, we identified specific adaptations in certain VRE PBPs that may facilitate the D-Ala-D-Lac utilization. We found that PBP1B, PBP2A, PBP3 showed changes, while PBP1A, PBP2B and PBP4 remained unchanged. Notably, a threonine-to-asparagine substitution at location 491 in PBP1B leads to a shift in substrate preference from D-Ala-D-Ala to D-Ala-D-Lac. Similar structural changes in PBP3 suggest that the presence of changed and unchanged PBPs within the same classes suggests compensatory interactions, indicating a teamwork among multiple PBPs. These insights into PBPs provide a deeper understanding of D-Ala-D-Lac utilization in VRE, may be used to develop new therapeutic agents to combat vancomycin resistance.

## 1 Introduction

The peptidoglycan in the bacterial cell wall is essential for maintaining cellular stability and protecting the bacterial cell from external osmotic pressures (Vollmer et al., 2008; Zapun et al., 2008a; Egan et al., 2015). Gram-positive bacteria have a substantially thicker peptidoglycan layer compared to gram-negative bacteria (Vollmer et al., 2008). Bacteria synthesize their peptidoglycan layer by employing penicillin-binding proteins (PBPs) (Vollmer et al., 2008; Zapun et al., 2008a; Egan et al., 2015). PBPs are membrane-associated proteins situated in the periplasmic region and involved in the extracellular final step of cell wall synthesis (Egan et al., 2020). The position of PBPs makes them a most appropriate target for antimicrobial agents.

PBPs are divided into two categories according to their molecular mass: The high molecular weight (HMW) and the low molecular weight (LMW) PBPs (Sauvage et al., 2008). HMW PBPs contribute to the polymerization of peptidoglycan and its integration into the existing cell wall structure. LMW PBPs exhibit hydrolase activity, modulating the composition and structure of cell walls by cleaving peptide bonds during cell growth and division (Goffin and Ghuysen, 1998; Born et al., 2006). Depending on the structure and function, HMW PBPs are further classified into class A or class B and LMW PBPs class C (Sauvage et al., 2008). Furthermore, PBPs are divided into three categories based on their enzymatic activity. Class A PBPs are the most common bifunctional PBPs and exhibit transpeptidase and glycosyltransferase activities that are responsible for the cross-bridges between peptides attached to neighboring polysaccharide chains. Class B PBPs are monofunctional proteins that possess only transpeptidase activity and Class C PBPs have carboxypeptidase and endopeptidase activities, which are responsible for peptidoglycan recycling (Vollmer and Bertsche, 2007; Sauvage et al., 2008; Pratt, 2008; Typas et al., 2011). Although the protein structures of PBPs vary according to bacterial species and its function, a PBP typically has three distinct domains: a N-terminal transmembrane domain anchoring the protein to the bacterial cell membrane, a nonpenicillin-binding domain acts as a flexible linker between an enzymatically functional C-terminal catalytic domain containing the catalytic serine (Sauvage et al., 2008; Pratt, 2008; Typas et al., 2011; Moon et al., 2018).

The catalytic serine is crucial for enzymatic activity and is strategically positioned within a distinct cleft known as the active cleft in the active site of PBPs. This region is prominently located within the transpeptidase domain and is defined by three highly conserved motifs. These motifs mark the active site and are involved in binding the substrate to the enzyme. *Motif I* (SxxK) contains the catalytic serine, which acts as a central component for initiating enzymatic reactions. *Motif II* [(S/Y)xN] is responsible for the protonation of leaving group of beta-lactams, while *motif III* (K(T/S)GT), with its distinctive sequence facilitates the binding between the enzyme and substrate (Ghuysen, 1991; Sauvage et al., 2008; Zapun et al., 2008b).

PBPs play a role in cross-linking the Lipid II stem pentapeptide (undecaprenyldiphospho-N-acetylmuramoyl-[N-acetylglucosamine]-L-alanyl-γ-D-glutamyl-L-lysyl-D-alanyl-D-alanine), which is the final intermediate in the growing peptidoglycan layer (Nakagawa and Matsuhashi, 1982; Tomioka et al., 1982; Egan et al., 2015). Additionally, the cross-linkage between two glycan chains is achieved by the removal of the final amino acid residue D-alanine from one of the pentapeptides by glycosylation, followed by transpeptidation to the other pentapeptide ( (Schleifer and Kandler, 1972; Rogers et al., 1980).

Vancomycin is a semisynthetic, glycopeptide type of antibacterial that is considered the ‘last resort’ treatment for multidrug-resistant infections caused by gram-positive bacteria (Moon et al., 2018). Vancomycin recognizes and binds specifically to the D-Ala-D-Ala residues at the ends of the pentapeptides; thus, inhibiting cross-linkage by preventing PBP binding. In vancomycin-resistant bacteria, peptidoglycan precursors are synthesized with ends consisting of D-Ala-D-Lac or D-Ala-D-Ser instead of D-Ala-D-Ala, which inhibits the binding of vancomycin (Boneca and Chiosis, 2003; Courvalin, 2005; Leclercq et al., 2017) because it has lower binding affinity to D-Ala–D-Lac and D-Ala-D-Ser than to the normal dipeptide (Bugg et al., 1991a). However, the identities of the PBP(s) that have the ability to bind to these new precursors (D-Ala-D-Lac or D-Ala-D-Ser) and continue to form cross-bridges in vancomycin-resistant isolates, are currently unknown. Moreover, it is not clear whether the binding abilities of existing PBPs to D-Ala-D-Lac or D-Ala-D-Ser are similar to that to D-Ala-D-Ala and whether this ability was acquired through mutations.

Vancomycin resistance is mediated by eleven gene clusters. The precursor ending with D-Ala-D-Lac is encoded by *vanA, B, D, F, I and M* clusters, whereas that of D-Ala-D-Ser is encoded by van*C, E, G, L* and *N* clusters (McKessar et al., 2000; Courvalin, 2005; Boyd et al., 2008; Xu et al., 2010; Lebreton et al., 2011). The most prevalent types of resistance globally are vanA and vanB, which are found on transposons Tn1546 (Arthur et al., 1993) and Tn1549 (Garnier et al., 2000), respectively. These types of resistance are primarily observed in *Enterococcus faecalis* and *Enterococcus faecium* (Cetinkaya et al., 2000).

The *vanA* operon contains *vanA, vanH* and *vanX*, as well as the regulatory genes *vanR* and *vanS*. The VanS serves as a sensor that is auto phosphorylated upon detecting vancomycin. This phosphate group is then transferred to VanR, which becomes activated and binds to two promoters in the *vanA* gene cluster as a transcriptional activator (Arthur et al., 1993, 1997; Cetinkaya et al., 2000). VanA is a ligase that produces D-Ala-D-Lac instead of D-Ala-D-Ala (Bugg et al., 1991b), whereas VanH is a D-lactate dehydrogenase that forms the D-Lac pool (Arthur et al., 1991; Bugg et al., 1991a). VanX is a D, D-dipeptidase that digests D-Ala-D-Ala specifically; it has no activity against D-Ala-D-Lac. These operons are involved in decreasing the binding affinity of antibiotics to peptidoglycan precursors, which in the case of D-Ala-D-Lac, results in an affinity reduction of approximately 1000-fold (Reynolds et al., 1994; Cetinkaya et al., 2000). Although the process of converting D-Ala-D-Ala to D-Ala-D-Lac is fully understood, the manner wherein PBPs can use these new types of pentapeptide end groups is unknown. The aim of this study was to investigate whether PBPs are modified and the potential role of these modifications in adapting to the use of D-Ala-D-Lac instead of D-Ala-D-Ala.

## 2 Materials and methods

### 2.1 Strains

Our experiments used five vancomycin-resistant and three vancomycin-susceptible *E. faecalis* strains, which were frozen stocks of clinical isolates. We identified the isolates to the species level using MALDI-TOF MS (Microflex LT; Bruker Daltonik GmbH, Bremen, Germany). For this purpose, bacteria from freshly grown colonies were smeared on the MALDI-TOF plate and formic acid solution was added on the smears, before evaluation with the instrument. The frozen stocks of clinical isolates were inoculated on Mueller–Hinton agar (MHA) (Dehydrated infusion from Beef 300.0 g/L; Casein hydrolysate 17.5 g/L; Starch 1.5 g/L., Agar 17 g/L) plates by single colony inoculation method and incubated at 37°C for approximately 24 hours. Single colonies were selected from the petri dishes and inoculated into Mueller Hinton broth (MHB) (5 ml, dehydrated infusion from Beef 300.0 g/L; Casein hydrolysate 17.5 g/L; Starch 1.5 g/L., pH: 7.3 ± 0.1) medium in 50 ml of tubes and incubated in a shaking incubator at 37°C with a speed of 180 rpm overnight. The bacterial suspension was measured at an absorbance of 600 nm (OD600) using a NanoDrop One spectrophotometer (Thermo Scientific, US), and the concentration was adjusted to approximately 0.9 ± 0.1. The vancomycin susceptibility of the strains was determined by the Kirby–Bauer disk diffusion method (Hudzicki, 2009). All VRE included in the study were identified as vanA-type based on the detection of *vanA* gene using specific primers: 5′AATACTGTTTGGGGGTTGCT3′ and 5′GCTTGACTAACTGGCGAACT3′ (Jelinkova et al., 2018).

### 2.2 DNA fingerprinting

The DNA of vancomycin-susceptible and -resistant *E. faecalis* strains were analyzed using pulsed-field gel electrophoresis (PFGE). Briefly, bacterial colonies were selected from MHA plates and inoculated into 5 mL of MHB medium. The bacteria were grown overnight at 37°C and then centrifuged at 10,000 x *g* for 2 minutes. The resulting pellet was washed twice with Solution-1 (10 mM Tris pH 8.0, 1 M NaCl) and resuspended in Solution-1. The final concentration of the bacterial suspension was adjusted to OD_620_ = 5.0. Bacterial cells were embedded in 0.15% low-melting agarose discs, which were prepared by dispensing 20 μL aliquots into parafilm-coated glass plates and cooling at −20°C for 5 minutes until solidified. To release bacterial DNA, the cell membrane of the embedded cells was lysed using lysis solution-2 (6 mM Tris pH 8.0, 1 M NaCl, 100 mM EDTA pH 8.0, 0.5% Na-lauryl sarcosine, 0.2% Na-deoxycholate, containing 5 µL/mL (10 mg/mL) RNase A, 5 µL/mL (20 mg/mL) lysozyme (Omega Bio-Tek, US) and 5 µL/mL (10 mg/mL) lysostaphin (ProSpec, Israel)) at 37°C for approximately 20 hours until the discs became completely transparent. The lysis solution was removed by sterile gauze, and the discs were incubated in Proteinase-K solution-3 (0.5 M EDTA pH 9.0, 1% sarcosyl containing 1mg/ml Proteinase-K) at 65°C for at least 18 hours to digest the residues. The DNA discs were washed five times using 10 mL of TE buffer (10 mM Tris pH 8, 1 mM EDTA pH 8) for 30 minutes each time. The DNA was digested with the restriction enzyme *SmaI* (New England Biolabs, UK) at 37°C for one hour. The digested DNA was then run in a 1% pulsed-field certified agarose gel using the PFGE CHEF DRII apparatus (Bio-Rad Laboratories, USA) for 18 hours at 6 V/cm, with pulse times of 5 seconds for the first switch and 35 seconds for the last switch. After electrophoresis, the PFGE gel was stained with ethidium bromide and imaged using the ChemiDoc MP Basic Imaging System (Bio-Rad Laboratories, US).

### 2.3 Data collection

The PBP gene sequences displayed in **Table 1** belong to the vancomycin-resistant *E. faecalis* V583 strain, which was the first such strain to have its whole genome sequenced (**Table 1**) (Paulsen et al., 2003). Furthermore, PBP amino acid sequences of selected complete genome-analyzed VRE and VSE strains were retrieved from the GenBank database for comparison with the PBP sequences of clinical strains (**Table 2**).

**Table 1:** Classification of penicillin-binding proteins of vancomycin-resistant strain *E. faecalis* V583.

	Class	Protein	Locus tag	Protein ID
HMW^a^	Class A	PBP1A	EF_1148	AAO80948.1
		PBP2A	EF_0680	AAO80501.1
		PBP1B	EF_1740	AAO81514.1
	Class B	PBP4	EF_2476	AAO82193.1
		PBPC/3	EF_0991	AAO82193.1
		PBP2B	EF_2857	AAO82549.1
LMW^b^	Class C	D-alanyl-D-alanine carboxypeptidase	EF_3129	AAO82807.1
		PBP/Serine hydrolase	EF_0746	AAO80564.1

a High Molecular Weight;

b Low Molecular Weight

**Table 2:** Information of the reference strains, vancomycin resistance status, protein accession numbers of PBP1B, PBP2A and PBP3.

Strain	Vancomycin resistance	Accession number	Protein ID. PBP1B	Protein ID. PBP2A	Protein ID. PBP3	Reference
*E. faecalis* V583	VRE	AE016830.1	AAO81514.1	AAO80501.1	AAO80797.1	Paulsen et al., 2003
*E. faecalis* ATCC 51299	VRE	JSES00000000.1	KGQ74720.1	KGQ75158.1	KGQ73849.1	Sung et al., 2015
*E. faecalis* ATCC 29212	VSE	CP008816.1	AIL04223.1	AIL05477.1	AIL03712.1	Minogue et al., 2014
*E. faecalis* D32	VSE	NC017316.1	AFO44358.1	AFO43389.1	AFO43695.1	Zischka et al., 2012
*E. faecalis* 62	VSE	CP003726.1	ADX80336.1	ADX79303.1	ADX79669.1	Brede et al., 2011

### 2.4 Primer designing, DNA isolation and amplification of PBP genes

The primers were designed with reference to the PBP genes presented in **Table 1** using Primer BLAST (The Basic Local Alignment Search Tool) online bioinformatics tool (Altschul et al., 1990). In order to enhance the precision of the Sanger sequencing method, the PBP genes, which are larger than 2000 bp, were amplified in three overlapping fragments.

Bacteria were disrupted by microbead homogenization in a lysis solution (TE buffer containing 5 µg/mL lysozyme and 10 µg/mL lysostaphin). Bacterial genomic DNA was purified using the Omega E.Z.N.A.^®^ Bacterial DNA Kit (Omega Bio-Tek, USA), according to the manufacturer’s instructions.

The amplification of PBP genes was conducted using isolated genomic DNA extracted from *E. faecalis* strains. The reactions were performed using the MyTaq HS DNA Polymerase (Bioline, US) with designed primers for each PBP gene. Polymerase chain reaction (PCR) mixtures were prepared in 50 µL volumes containing following components: 5X MyTaq Buffer (10 µL, 5 mM dNTPs, 15 mM MgCl_2_), MyTaq Hot Start DNA Polymerase (1 µL), forward and reverse primers (1 µL, 20 µM each), template DNA as required (50 ng/µL), adjusted to 50 µL with nuclease-free water. PCR was performed on a T100 Thermal Cycler (Bio-Rad, US) using the following conditions: initial denaturation at 95°C for 3 minutes, denaturation at 95°C for 15 seconds, annealing at the optimal temperature for each primer pairs for 15 seconds, extension at 72°C for 15 seconds for 34 cycles of amplification and final extension at 72°C for 5 minutes. (The detailed information of primers and annealing temperatures is provided in **Supplementary Table 2**).

The amplified PBP genes were analyzed via agarose gel electrophoresis to confirm the expected band sizes and visualized under UV light following staining with ethidium bromide. The confirmed DNA bands were extracted from the gel. Nucleotide sequences were read using the Sanger sequencing method with the service of GATC Biotech AG in Germany.

### 2.5 Bioinformatics analysis

The PBP gene sequences were converted to amino acid sequences according to reading frames using EXPASY translate tool (Swiss Institute of Bioinformatics, Switzerland) and aligned and compared using the ClustalW algorithm (Thompson et al., 1994) in Jalview software (Waterhouse et al., 2009). The conserved motifs were analyzed and the active site of each PBP was identified by determining the positions of the active serine (S) residues (**Table 3**).

**Table 3:** Amino acid residue changes detected in penicillin-binding proteins.

Protein	Substitutions	Active Site indicating motif I	Catalytic Serine Location
PBP1A	None	STVK	417
PBP1B	T491N	STIK	450
PBP2A	K336G	SSLK	402
PBP2B	None	SVVK	406
PBP3	N326D, L345I, S350T, A353S, I354M, L359Q, S364L, I368V, T372Q, T376V, I378V, V383Y, G384T, N385R, K387N, D388G, T390E, S391T, T393N, N408K, R412K, G415D, D416E, V419M, S429T, T436S, S437G, H439S, T440A, D445G, N446T, T447N, I448F, N453M, G460A	STIK	343
PBP4	None	STFK	424

### 2.6 Modeling and docking studies of the PBPs

PBPs of VRE and VSE were modeled using the SWISS-MODEL Homology Modeling (Swiss Institute of Bioinformatics, Switzerland) online tool by comparing amino acid sequences to previously modeled PBPs present in the Protein Data Bank (PDB) (Waterhouse et al., 2018). The ligand binding dynamics were analyzed in UCSF Chimera version 1.16 (Pettersen et al., 2004) by using AutoDock Vina (Trott and Olson, 2009; Eberhardt et al., 2021) software. Binding site was defined by the detection of the active serine containing motif I. A rectangular docking box was formed around the transpeptidase domain of the protein, and the ligand was positioned inside the docking box, close to the active site. The dimensions of the docking box, approximately 46x46x51 Å, were defined to encompass the whole transpeptidase domain where the active site is located, ensuring that the ligands had sufficient space to explore binding conformations. This ensures the active site is adequately covered while avoiding non-relevant areas of the protein. The ligands were initially at a distance of approximately 30 Å from the active serine, allowing for unbiased exploration of potential binding poses during docking simulations. This placement was adequate to enable both interactions with the active site and the movement of the ligands without restriction. The ligands L-Lys-D-Ala-D-Lac and L-Lys-D-Ala-D-Ala were prepared using ChemDraw (PerkinElmer Informatics, US) in pdbqt format. The 3D binding patterns and affinities of each ligand on both VSE and VRE PBPs were determined and compared. The three-dimensional (3D) models were visualized using Chimera X version 1.3 (Goddard et al., 2017; Pettersen et al., 2020).

## 3 Results

In the VRE strains included in this study, no substitutions were detected in PBP1A, PBP2B and PBP4, in contrast, substitutions leading to structural changes were detected in PBP1B, PBP2A and PBP3. In the comparative analyses conducted with the PBPs of VRE strains from the GenBank database, the same substitutions were identified in PBP1B and PBP2A, aligning with the findings of this study. However, the thirty-five substitutions detected in PBP3 in the study strains were not observed in the PBP3 of VRE strains from the database; instead, these strains exhibited an equal number of amino acid changes in PBP2B. A further finding was that the amino acid sequences of the strains in the GenBank database were not different from those of the strains analyzed in this study.

**Figure 1:**
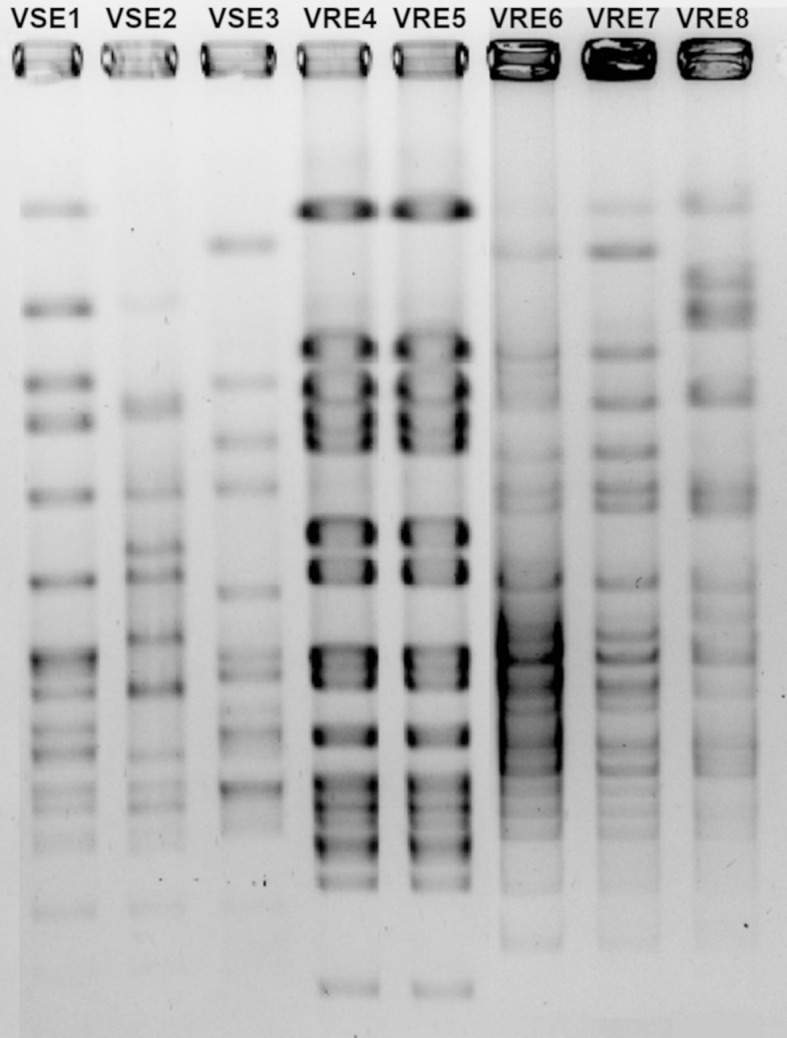
Pulsed-field gel electrophoresis gel image of vancomycin-resistant (VRE) and-susceptible (VSE) *E. faecalis* strains.

Moreover, among the five VRE strains, VRE4 and VRE5 as well as VRE6 and VRE7, shared similar DNA fingerprint patterns, indicating that VRE4 and VRE5, are the same strains isolated from different patients, as are VRE6 and VRE7. However, VRE4-5, VRE6-7 and VRE8 differed in their DNA fingerprint patterns, indicating that three different strains of VRE were included in this study. All three VSE strains used to determine PBP sequences differed in their DNA fingerprint patterns and were thus different strains (**Figure 1**).

**Figure 2:**
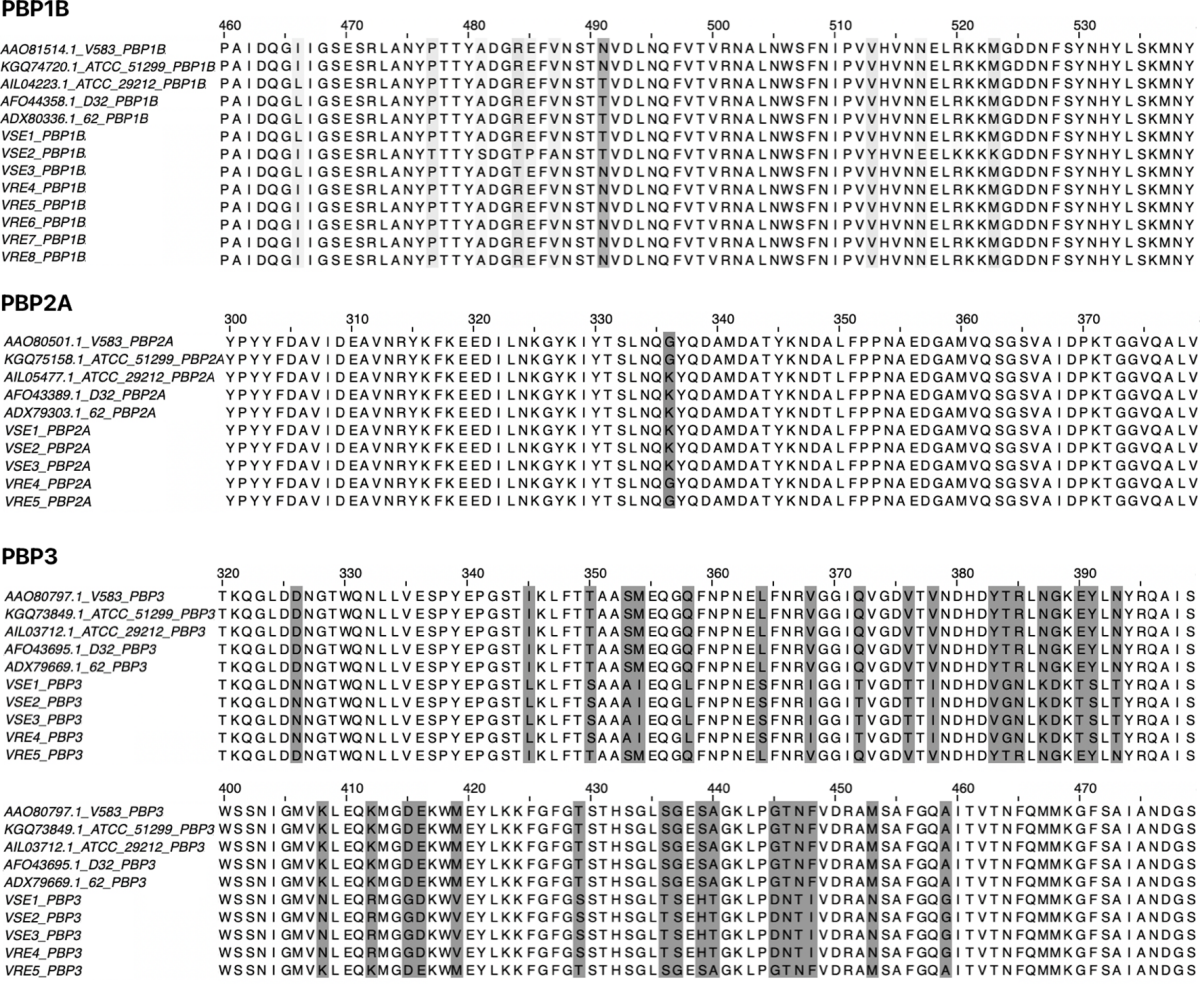
Amino acid alignment of PBPs (PBP1B, PBP2A and PBP3) from vancomycin-resistant and vancomycin-susceptible *E. faecalis* strains. Amino acid sequences of PBPs were aligned to identify substitutions in VRE and VSE strains. The alignment includes four VRE strains (V583 and ATCC 51299 from the GenBank database, and clinical isolates VRE4 and VRE5) and six VSE strains (ATCC 29212, D32 and 62 from the GenBank database, and clinical isolates VSE1, VSE2 and VSE3). For PBP1B, additional VRE strains (VRE6, VRE7 and VRE8) were included to increase sample size. Amino acid differences between resistant and susceptible strains are highlighted, with emphasis on conserved substitutions and their positions in aligned PBPs.

**Figure 3:**
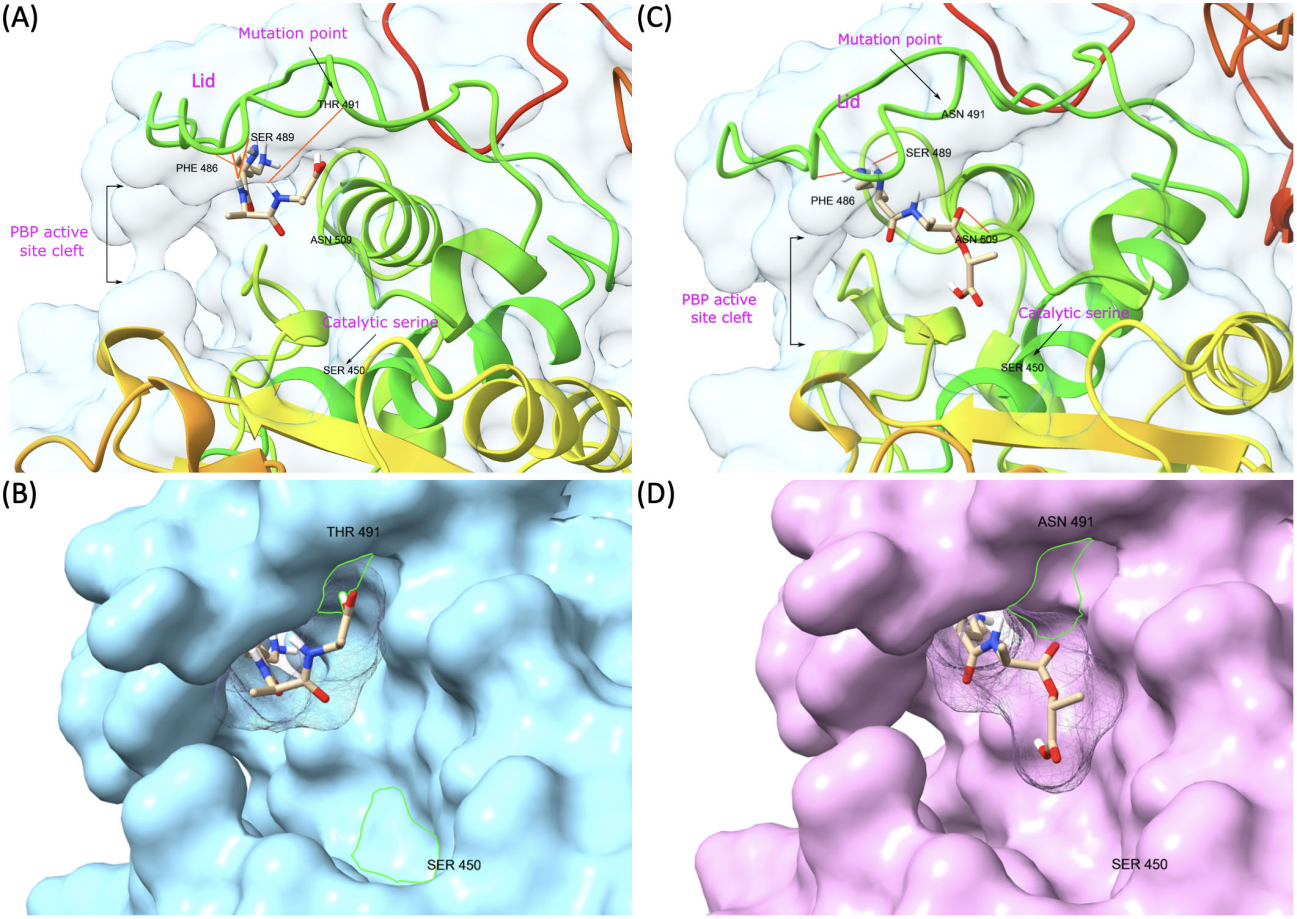
Representative model of PBP1B showing mutation point and ligand binding in the active site. **(A)** VSE PBP1B and the native ligand (L-Lys-D-Ala-D-Ala) complex. **(B)** Surface representation of VSE PBP1B and docked native ligand. **(C)** Asn-modified VRE PBP1B and changed ligand (L-Lys-D-Ala-D-Lac) complex. **(D)** Surface representation of VRE PBP1B and docked altered ligand.

**Figure 4:**
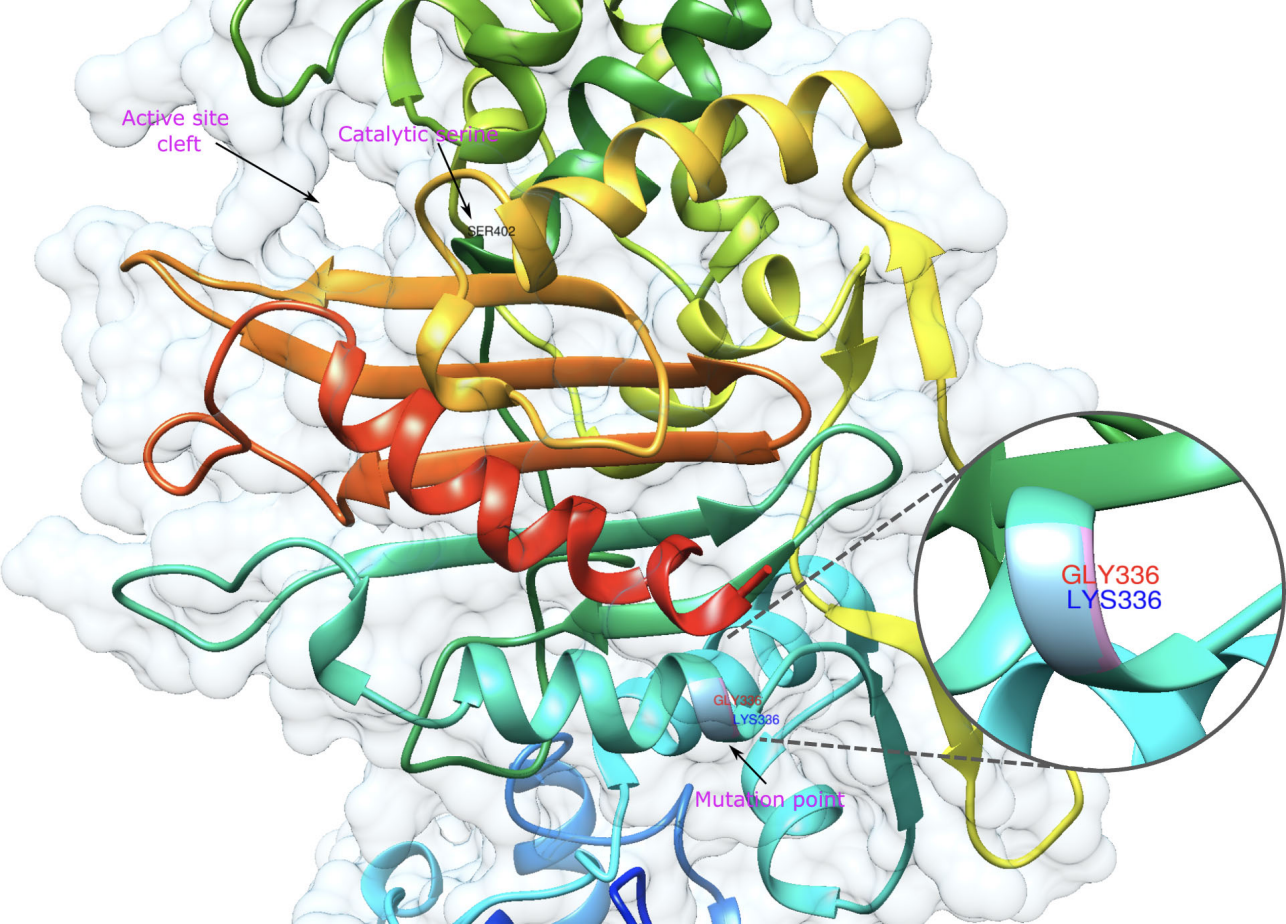
Representative model of PBP2A showing mutation point wherein VRE and VSE strains differ. The amino acids in the mutation point are shown in the zoomed-in area and are labelled in red and green for VRE and VSE, respectively.

**Figure 5:**
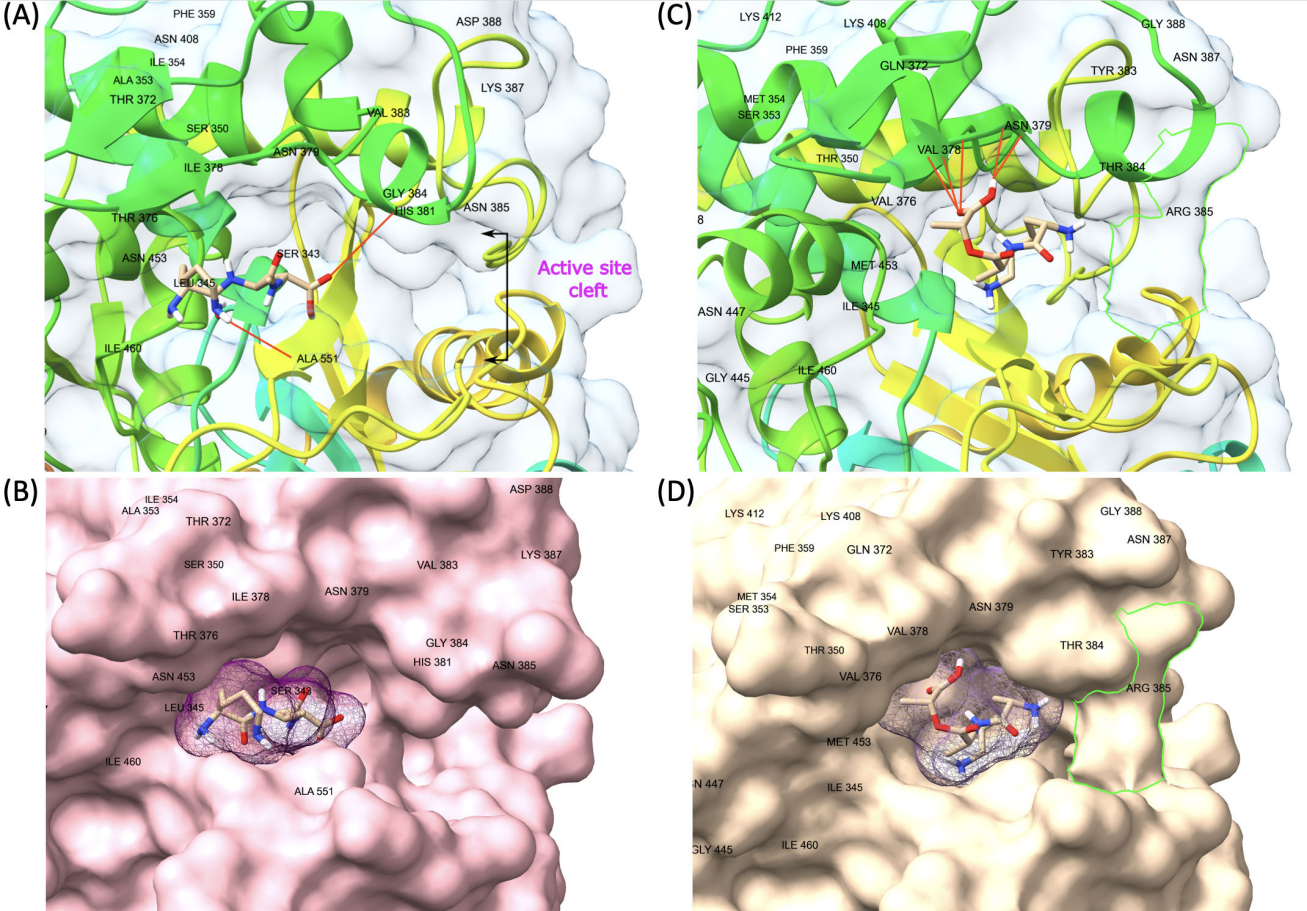
Representative model of PBP3 showing changes on transpeptidase domain. The green line **(D)** is framing a changed region that creates a barrier-like structure at the entrance of the active cleft of VRE PBP3 with the replacement of asparagine by arginine at residue 385. **(A, B)** VSE PBP3 and L-Lys-D-Ala-D-Ala complex and surface representation. **(C, D)** VRE PBP3 and L-Lys-D-Ala-D-Lac complex and surface representation.

PBP1B had a different amino acid, asparagine (Asn or N), at codon 491 in all VREs, including the strains, the sequence data of which were obtained from the GenBank database. There was a threonine (Thr or T), at this position, in all VSE analyzed except VSE3, which is identical with VRE strains at this site (**Figure 2**). This amino acid is located inside the active cleft, opposite the catalytic serine (**Figures 3A, C**). This change from threonine to asparagine replaces the negative polar group (OH) with a positive polar group (NH_2_) on the side chain, which decreases the binding affinities to L-Lys-D-Ala-D-Ala and penicillin (**Table 4**). Similarly, in PBP2A, we found VSE and VRE had a lysine (Lys or K) or glycine (Gly or G), respectively, at codon 336 (**Figure 2**) and the structural position of this substitution is shown in the protein model (**Figure 4**). PBP3 shared the same amino acid sequences in all VSE and VRE4. However, in VRE5, we found a major change affecting 35 amino acids (**Figure 2**) located in the region forming the active site of the transpeptidase domain of PBP3 (**Figure 5**).

**Table 4:** The results of receptor binding affinity analysis for both the native and modified ligands.

Receptor	Ligand	Score* (kcal/mol)
VSE PBP1B	L-Lys-D-Ala-D-Ala	−6.2
VRE PBP1B	L-Lys-D-Ala-D-Ala	−5.5
VSE PBP1B	L-Lys-D-Ala-D-Lac	−5.6
VRE PBP1B	L-Lys-D-Ala-D-Lac	−6.1
VSE PBP2A	L-Lys-D-Ala-D-Ala	−6.2
VRE PBP2A	L-Lys-D-Ala-D-Ala	−6.1
VSE PBP2A	L-Lys-D-Ala-D-Lac	−5.5
VRE PBP2A	L-Lys-D-Ala-D-Lac	−6.1
VSE PBP3	L-Lys-D-Ala-D-Ala	−5.4
VRE PBP3	L-Lys-D-Ala-D-Ala	−5.5
VSE PBP3	L-Lys-D-Ala-D-Lac	−5.7
VRE PBP3	L-Lys-D-Ala-D-Lac	−5.6

*Scores refer to binding free energy values, where a lower score indicates higher binding stability.

## 4 Discussion

Over the past few decades, VRE has emerged as a critical agent of nosocomial infections. In 2019, VRE were responsible for 30% of hospital-acquired infections and related deaths in the USA (2019 Antibiotic Resistance Threats Report, 2024). A recent study by the European Centre for Disease Prevention and Control reported an approximately twofold increase in VRE infections between 2007 and 2015, followed by a 2.5-fold increase from 2016 to 2020 (Merk et al., 2022). Furthermore, the emergence of vancomycin resistance in methicillin-resistant staphylococci (MRSA) represents a significant cause for concern (Kanlidere et al., 2018; Lade and Kim, 2023).

Understanding the mechanisms of vancomycin resistance is crucial for developing new therapeutic strategies to treat serious infections caused by VRE. Although the changes in the glycan side chain responsible for vancomycin resistance are well established (Bugg et al., 1991a; Arthur et al., 1992; Wang et al., 2018; Stogios and Savchenko, 2020), the question of how PBPs that typically utilize D-Ala-D-Ala as a substrate during transpeptidation can adapt to use D-Ala-D-Lac remains unclear so far. Previous research has demonstrated that specific amino acid substitutions in PBPs are capable of developing resistance to β-lactam antibiotics (Jakubu et al., 2024). In this study, we investigated potential amino acid changes in the PBPs of VRE that may allow for the accommodation of these modified peptide side chains during transpeptidation.

We discovered mutations in three of the six HMW PBPs present in VRE, which led to alterations in the 3D structure of these proteins. The changed PBPs were PBP1B, PBP2A and PBP3, while the sequences of PBP1A, PBP2B and PBP4 remained identical to those found in VSE. Particularly, the change from threonine to asparagine at position 491 in PBP1B results in a shift from a negatively to a positively polar group. Threonine residues, which are commonly found in functional centers of proteins, possess a reactive hydroxyl group capable of forming hydrogen bonds with various polar substrates. In contrast, asparagine, another polar amino acid, is typically located on the protein surface where it interacts with an aqueous environment and frequently involves in active or binding sites. Its polar side chain is highly reactive, readily interacting with other polar or charged atoms. Our 3D model of PBP1B revealed that this amino acid substitution occurs at the active site of the transpeptidase domain. Molecular docking analysis further showed that the Asp-modified PBP1B had a higher affinity for D-Ala-D-Lac, whereas the wild-type PBP1B showed a preference for D-Ala-D-Ala (**Table 4**). Since D-Lac moiety contains a negatively charged group which is expected to be attracted to the positive polar group of asparagine, this may explain the affinity increase to D-Ala-D-Lac by this amino acid change. Furthermore, the analysis of structural models indicates that the Asn substitution induces minor conformational changes in the binding site, resulting in the formation of a more compacted region (**Figure 3D**) in comparison to the presence of Thr (**Figure 3B**). This change may optimize the active cleft for D-Ala-D-Lac binding. These results suggest a selective adaptation in which VRE develops mutations that provide a functional advantage in the presence of antibiotics targeting cell wall synthesis. This finding aligns with the observed tendency of PBPs in VRE to utilize D-Ala-D-Lac moieties instead of D-Ala-D-Ala.

A substitution from lysine to glycine at position 336 of PBP2A was identified (**Figure 4**). The position of this substitution, while relatively distal to the active site, is proximal to the transmembrane domain of PBP2A. Binding affinity scores showed similar values for D-Ala-D-Ala in both VRE and VSE PBP2A (-6.1 and -6.2 kcal/mol, respectively). However, VSE PBP2A exhibited a reduced affinity for D-Ala-D-Lac (-5.5 kcal/mol) compared to VRE PBP2A (-6.1 kcal/mol) (**Table 4**). The substitution of Lys to Gly at this position may have effects on flexibility and stability of the protein in the proximity of the membrane interface, with the potential to mediate substrate accessibility.

Another significant change affecting 35 amino acids surrounding the enzyme’s active site was found in the transpeptidase domain of PBP3 of VRE5 strain. These substitutions result in significant structural changes in the protein. A noteworthy change was observed at position 385 from asparagine (Asn or N) to arginine (Arg or R) which formed a barrier-like structure at the entrance of the active cleft. Although this barrier-like structure appears to narrow the active cleft and is supposed to have a negative impact on the substrate binding, no significant difference in binding affinity values were observed in both ligands. This result may indicate that the active cleft, even in its narrowed state, possesses sufficient opening to accommodate such small molecules as D-Ala-D-Ala or D-Ala-D-Lac. Furthermore, substitutions indicated exceptions between some VRE and VSE strains. The VSE3 strain was identified to possess the same mutation at position 491 in PBP1B as resistant strains and a similar exception was also observed for VRE4 in PBP3, in line with the susceptible strains. The presence of identical mutations between VRE and VSE strains may be attributed to horizontal gene transfer or spontaneous mutation. Despite the similarity in DNA fingerprinting patterns observed between VRE4 and VRE5, it was noted that a large number of mutations were observed only in VRE5 in PBP3. This finding can be explained by the absence of *SmaI* restriction enzyme cutting site, which was used in DNA fingerprint analysis, in both wild type and mutated sequences of PBPs. It has been demonstrated through protein modeling that these changes resulted in the formation of a barrier-like structure at the entrance of the active cleft, which was not previously present. These conformational changes may have created an allosteric site in the protein. It would be essential to confirm this information through further structural and functional analyses.

The production of D-Ala-D-Lac by the genes of van operon is under very strict control. Because D-Ala-D-Ala units are not converted to D-Ala-D-Lac in the absence of vancomycin, changes in only three of six HMW PBPs of VRE allow for cell wall synthesis to proceed similarly to that of unchanged PBPs. The observation that a PBP within class A is changed while another remains unchanged, with similar patterns in class B, suggests a coordinated compensatory mechanism among multiple PBPs. It is logical to think that vancomycin susceptible enterococci would prefer D-Ala-D-Ala ends of the pentapeptides like many other species of bacteria as these groups are selected as optimal groups to make cross-bridges in peptidoglycan synthesis during evolution of bacteria for millions of years. D-Ala-D-Lac groups emerged due to the selective pressure of vancomycin. Any such change creates a disadvantage for bacterial growth however bacteria have no other choice in the presence of vancomycin. Therefore, it is advantageous for bacteria to modify the end of pentapeptides to D-Ala-D-Lac groups in the presence of vancomycin. If all PBPs were mutated to adapt to this change the bacteria would lose its ability to produce cell wall at its original speed. However, by keeping half of their PBPs at their original conformation they can multiply as other vancomycin susceptible enterococci in the absence of vancomycin. These adaptive changes among PBPs reveal insights into the cooperative roles of different PBPs in cell wall synthesis. Notably, D-Ala-D-Ala may remain the preferred substrate in the absence of vancomycin, highlighting a context-dependent functionality of these proteins.

Our results provide new insights into the molecular mechanisms underlying D-Ala-D-Lac utilization during transpeptidation. This information may be useful for developing novel therapeutic agents to combat VRE. Since β-lactam antibiotics are analogues of D-Ala-D-Ala, our study revealed their reduced affinity for mutated PBPs in VRE. Therefore, the design of D-Ala-D-Lac analogues could potentially inhibit the activity of mutated PBPs, offering a promising strategy for the treatment of VRE infections.

## Data Availability

The raw data supporting the conclusions of this article will be made available by the authors, without undue reservation.
