# Supplementary material for: Discovery of amino acid substitutions in penicillin-binding proteins associated with adaptation to D-Ala-D-Lac in vancomycin-resistant Enterococcus faecalis
Source: Front Cell Infect Microbiol. 2025 Feb 11;15:1522114. doi: 10.3389/fcimb.2025.1522114 (PMC11850342; doi:10.3389/fcimb.2025.1522114)
Supplement: Supplementary file 1 [file DataSheet1.pdf]

Supplementary Material

1 Supplementary Tables

Table 1. Antimicrobial susceptibility test results of *E. faecalis* clinical isolates.

**Strain Tag**	**VSE1**	**VSE2**	**VSE3**	**VRE4**	**VRE5**	**VRE6**	**VRE7**	**VRE8**
**Sample**	Urine	Urine	Urine	Skin fistula	Rectal swab	Urine	Rectal swab	Blood
**Ciprofloxacin**	S	S	R	R	S	R	ND	ND
**Trimethoprim-sulfamethoxazole**	IS	IS	IS	R	R	ND	ND	ND
**Vancomycin**	S	S	S	R	R	R	R	R
**Tigecycline**	S	S	S	S	S	ND	S	S
**Linezolid**	S	S	S	S	S	S	S	S
**Erythromycin**	R	R	R	R	R	ND	ND	ND
**Penicillin G**	R	R	R	R	R	ND	ND	ND
**Teicoplanin**	S	S	S	R	R	R	R	R
**Ampicillin**	R	R	R	R	R	R	S	S
**Clindamycin**	R	R	R	R	R	ND	ND	ND
**Tetracycline**	R	R	R	R	R	ND	ND	ND
**Gentamycin (High-level)**	S	S	R	R	R	R	R	R

S: Sensitive, IS: Intermediate sensitive, R: Resistant, ND: No data.

Table 2. Primers for amplification of PBPs.

Primer Tag	Sequence	Primer Length (bp)	Annealing Temperature (◦C)	Product Length (bp)
PBP1a Fw	TTATGCTGCTTTATTTTCATCTGG	24	54	2337
PBP1a Rv	ATGCCAACCGCAAATTCAG	19	56	2337
PBP1a_1 Rv	GCTTATGCGGCTTTCG	16	54	801
PBP1a_2_Fw	GTTATAAATCCCGTTATTGGCG	22	55	801
PBP1a_2_Rv	GTTGTACTTTACACAATGTACG	22	52	801
PBP1a_3_Fw	CGGCTTTCGAGATTTTTTATTG	23	55	817
PBP1b Fw	ATGTATCATTTTATTGAGGTGAAGC	25	54	2412
PBP1b Rv	GCGAGTTAATTATTATTCTTTTTATTATCG	30	53	2412
PBP1b_1_Rv	GACAAATCGTCTTTCAAGGC	20	54	821
PBP1b_2_Fw	CACGCCCTATACTAACACTG	20	54	852
PBP1b_2_Rv	GCATCTGAAGCTGGATAGTTC	21	55	852
PBP1b_3_Fw	CACTACTTAAGTAAAATGAACTATCC	26	52	824
PBP2a Fw	ATGGACAATCTTAAACAATTTTTAGT	27	53	2187
PBP2a_Rv	CTAATTTCCTAATAAGCCTCCGA	23	53	2187
PBP2a_1_Rv	CGACCATTAGTTGTAAAACGG	21	55	796
PBP2a_2_Fw	CATTGACAATGCCACAGC	18	53	752
PBP2a_2_Rv	CACCCGTTTGTAATCCTCC	19	54	752
PBP2a_3_Fw	CGGTACTATGGTTTAGCCC	19	55	729
PBP2b Fw	TTATTTTTTGTACATTTCCATATACGC	27	53	2136
PBP2b Rv	GAGGTATGAAGAAAAACTCATTTATAG	27	52	2136
PBP2b_1_Rv	CGGAACAACCATGCAATTAAC	21	52	790
PBP2b_2_Fw	GAATATTCTAACGCTTGCTCC	21	55	753
PBP2b_2_Rv	GAATGAAGGGGTAACAGAAGG	21	53	753
PBP2b_3_Fw	GCTGTGTGTTCACCAATTG	19	54	694
PBP3 Fw	TGAGTAAAAGACATAAATTTAAACAATTC	30	52	2229
PBP3 Rv	TTATTCTGTGCCTTCTAAAGTCAC	24	53	2229
PBP3_1_Rv	CAAAATACACACGGCCATC	19	55	727
PBP3_2_Fw	GGGACTAGAACAGACCTATAATG	23	54	782
PBP3_2_Rv	GAGGTTGCAACATTGAACC	19	54	782
PBP3_3_Fw	CCAAATGATGAAAGGTTTCTC	21	53	835
PBP4 Fw	TTATTTAATGGTTGCTTCTAAGTAATC	27	52	2048
PBP4 Rv	TTTATTACGATTGCTTCGTTCCAT	24	55	2048
PBP4_1_Rv	CATTGGTTTAGATGCAGGG	19	53	748
PBP4_2_Fw	CTAGCTCTTCATCTGGCTTC	20	55	714
PBP4_2_Rv	CAGCCAATATCAAAGCTTTTAGTG	24	54	714
PBP4_3_Fw	CCTTGGCTTAACTTTTGATTG	21	53	698

Fw: Forward primer, Rv: Reverse primer
